# Housing matters: Experimental variables shaping metabolism in obese mice

**DOI:** 10.1016/j.molmet.2025.102190

**Published:** 2025-06-18

**Authors:** Béatrice So-Yun Choi, Jacob Bak Holm, Asker Brejnrod, Even Fjære, Zhongkui Xia, Marie-Louise Allingbjerg, Ida Søgaard Larsen, David Møbjerg Kristensen, Morten Dall, Lene Secher Myrmel, Janne Koch, Niels Banhos Danneskiold-Samsøe, Otto Kalliokoski, Jonas T. Treebak, Liang Xiao, Axel Kornerup Hansen, Helle Sørensen, Lise Madsen, Manimozhiyan Arumugam, Karsten Kristiansen, Benjamin A.H. Jensen

**Affiliations:** 1 Department of Biomedical Sciences, Faculty of Health and Medical Sciences, University of Copenhagen, Denmark; 2 Laboratory of Genomics and Molecular Biomedicine, Department of Biology, Faculty of Science, University of Copenhagen, Denmark; 3 Cmbio, Copenhagen, Denmark; 4 Novo Nordisk Foundation Center for Basic Metabolic Research, Faculty of Health and Medical Sciences, University of Copenhagen, Denmark; 5 Institute of Marine Research Bergen, Norway; 6 Institute of Metagenomics, BGI-Shenzhen, Shenzhen, China; 7 Section of Biomedicine, Department of Veterinary and Animal Sciences, Faculty of Health and Medical Sciences, University of Copenhagen, Denmark; 8 Data Science Lab, Department of Mathematical Sciences, University of Copenhagen, Denmark; 9 Department of Clinical Medicine, University of Bergen, Norway

**Keywords:** Reproducibility, Animal models, High-fat diet, Housing conditions, Thermoneutrality, Sex differences

## Abstract

Diet-induced obesity in mice is an important model for investigating host–diet interactions as well as dietary and pharmacological treatments of metabolic diseases. Experimental reproducibility is, however, a recurrent challenge. To determine key controllable experimental drivers of mouse metabolism, we distributed 338C57BL/6JBomTac mice (males and females) into six research units across two countries, divided them into a variety of housing conditions (i.e., diets, cage types, temperatures, group-housing vs. single-housing) and kept 26 reference mice at the vendor. We applied linear mixed models to rank the influence of each variable on metabolic phenotype (i.e., body weight gain, glucose intolerance, liver, and visceral adipose tissue weight). Group-housing was the most potent driver of metabolic dysfunction apart from sex and diet. Accordingly, single-housed mice exhibited reduced weight gain (∼50%), increased energy expenditure, and diminished respiratory exchange ratio concomitant with improved glucose tolerance (∼20%) compared to their group-housed counterparts. Our results may aid in clarifying the impact of experimental design and promote rational, transparent reporting to increase reproducibility.

## Highlights

• Group-housing exacerbate metabolic dysfunction in both sexes.
• Sex differences significantly impact diet-induced metabolic responses.
• Ambient temperature has minimal impact on metabolic outcomes.
• Single-housing reduces obesity by boosting diurnal energy expenditure.

## Graphical abstract

**Figure:**
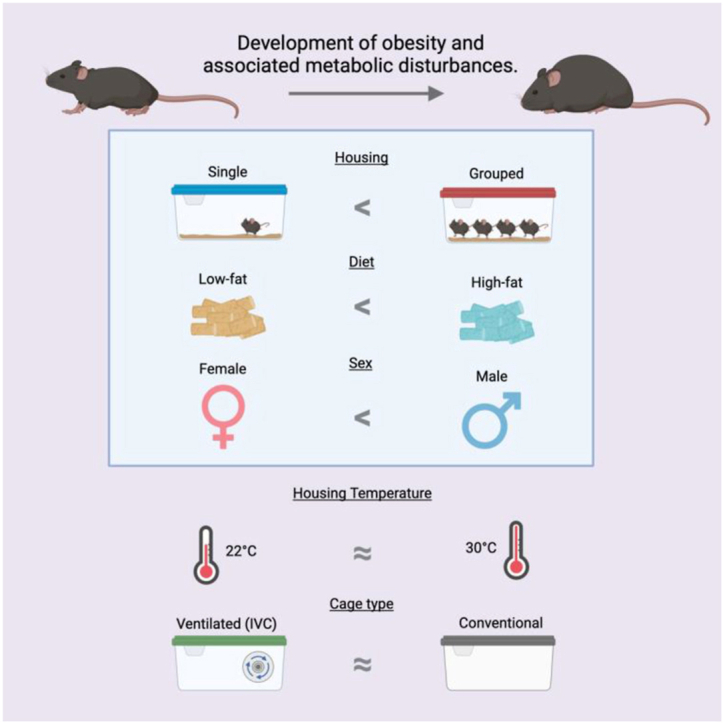

## 1 Introduction

The quest for reliable and reproducible preclinical research is a pressing concern in biomedical science, particularly in the context of diet-induced obesity (DIO) studies. With nearly 90% of all drugs entering clinical trials failing to reach consumer markets [1], the need to address preclinical translatability and experimental reproducibility is paramount [2,3]. This challenge is exacerbated by the low reproducibility rates in biomedical research, often cited as around 50%, which significantly delays the development of potentially lifesaving therapies [4]. To mitigate these challenges, it is essential to replicate key findings before publication, avoid overinterpretation of own research results, apply thorough reporting of methodology and experimental conditions, release raw data to public repositories, and mitigate statistical heterogeneity [2].

While rodent models of DIO are crucial for understanding metabolic diseases, they are highly susceptible to experimental variables affecting key outcomes, hence limiting generalizability of reported findings. Well-documented variables confounding biological interpretations include the choice of *i*) reference diet, *ii*) obesogenic diet, *iii*) sex, *iv*) mouse vendor, and *v*) mouse substrain [[5], [6], [7], [8], [9], [10], [11]]. Less systematically characterized variables include group versus single housing and ambient temperature [[12], [13], [14], [15]]. Mentioned variables are traditionally studied in silos, hence putting substantial strains on our mechanistic understanding of the interactions between these factors as well as their individual contribution to experimental biases.

In this study, we aimed to elucidate the hierarchy of these variables in DIO research, providing a framework to reduce intra- and inter-laboratory variability and enhance reproducibility. We hypothesised that housing conditions, which are notoriously underreported, significantly impact mouse behavior and phenotypic appearance, thus influencing DIO outcomes.

**Figure 1:**
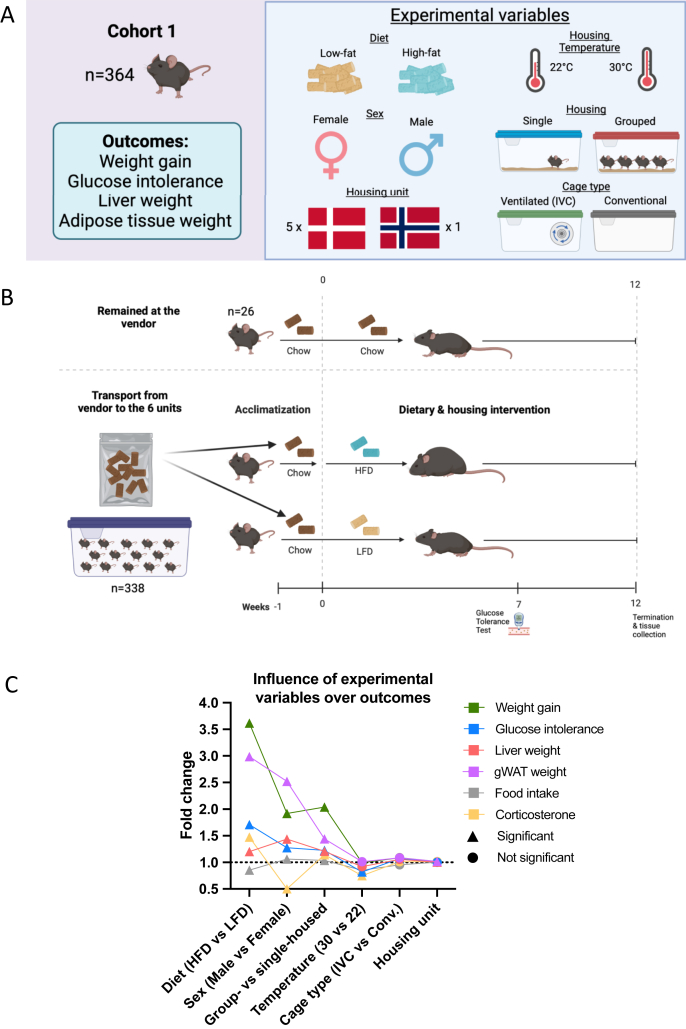
**Experimental design and the main effect of experimental variables.** A: Visual representation of the different experimental variables and outcomes measured. B: Experimental timeline. Mice were shipped to the six different units simultaneously, with a bag of food from the vendor for acclimatization in each unit. C: Model estimates from linear mixed models as the projected fold change. For housing unit, estimate for the average fold change compared to a typical unit. Each line represents a phenotypic outcome with experimental variables depicted on the x-axis. Circles indicate no significant effect of the variable over the outcome and triangles indicate significance.

To investigate the effects of these factors on DIO, we employed linear mixed models (LMM) on log-transformed data from 338C57BL/6JBomTac mice (260 males and 78 females) across six experimental units in two countries (Figure 1A). Additionally, we kept 26 mice (13 males and 13 females) at the vendor's breeding unit. Our analysis quantified the impacts of sex, diet, and housing conditions over 12 weeks.

By confirming that single-housed mice are broadly protected against DIO through an independent cohort focusing on activity, energy expenditure, and respiratory exchange, we highlight critical yet controllable experimental variables that can improve reproducibility.

## 2 Material and methods

### 2.1 Mice and ethics

All 364C57BL/6JBomTac mice (273 males and 91 females) from the first experiment were bred and delivered from unit EBU151, Taconic Biosciences, Denmark, and delivered at 6–7 weeks of age. Twenty-six reference mice (13 males and 13 females) were kept on a chow diet at the vendor's breeding unit. Upon delivery, mice were randomly assigned to their experimental cages and acclimatized for 8 days prior to the start of the experiment. All mice were packed, shipped, and delivered to their respective unit within the same day and fed batch-matched chow diet delivered by the mouse vendor i.e. “meal packs”, until the experiment started.

C57BL/6JBomTac mice (37 males) used for the follow-up experiment, designed to explore potential differences in energy expenditure between single- and group-housed mice, were bred and delivered from the same breeding unit as mice from the original experiment, but one year later. In this experiment, we either single- (n = 5) or group-housed mice (n = 8 cages with n = 4 mice per cage). In each of the 8 cages with group-housed mice, we randomly selected one representative mouse from the start of the experiment and followed this mouse throughout. The remaining three mice per cage served as cage mates and were used for other experimental purposes at the conclusion of this protocol. Food intake, energy consumption, and energy expenditure were measured on a cage basis and used to estimate an average consumption per mouse.

All experiments were conducted in accordance with the EU directive 2010/63/EU as approved by the Danish Animal Experiments Inspectorate (#2014-15-2934-01,027). Mice were kept under specific pathogen-free conditions at 22 °C (T_22_) or 30 °C (T_30_) ± 1°, as indicated, and humidity to 50%, ±5%. in a 12 h light/dark cycle (6AM–6PM) with *ad libitum* access to food and water. ARRIVE guidelines have been followed and described in this manuscript. Cohorts of mice were unblinded to the handlers as it is not possible to blind group vs single-housing and no mice were voluntarily excluded from analysis unless the data was missing due to, for example, termination of the animal [16].

### 2.2 Housing, diets, and experimental setup

Mice were randomly assigned to the six experimental units (n = 338) or the vendor's breeding unit (n = 26) and then randomly assigned to single- (n = 5 per condition) or group-housing (n = 8 per condition, divided into two cages of 4 mice per cage) in either conventional open (n = 130) or individually ventilated (IVC) Type II cages (n = 182) (both conventional and IVC had the following dimensions: L 36.5 cm × B 20.7 cm × H14.0 cm, Techniplast, Italy) except for unit 4, where mice were housed in disposable IVC cages (n = 52) (L 31.5 cm × B 20 cm × H 12.0 cm; Innovive, CA, USA). Please consult Supplementary Table 1 and Figure 1 for schematic overview.

All cages, water, water bottles, and feed were changed weekly. Weight and feed intake were registered at the day of cage change, ensuring minimal handling of the animals. At all times, male mice were handled before female mice to minimize stress and fighting behavior. To further minimize experimental variation, all feed (Ssniff Spezialdiäten, Germany), bedding (Tapvei, Finland), nesting material (Envirodri, Brogaarden, Denmark), red semi-transparent plastic shelters (“JAKO”; Molytex, Denmark), and gnawing sticks (Tapvei, Finland) were batch-matched; i.e. all components were collected in one unit and from there distributed to the other experimental units.

Mice were fed the standard chow diet used at Taconic Biosciences (Altromin 1324, Brogaarden, Denmark) from weaning until the experiment started. To ensure a stable baseline and to minimize the confounding influence of batch differences between chow diets, all mice were supplied with batch-matched diets from Taconic Biosciences before shipment to experimental units. After 8 days of acclimatization at the experimental units, mice (n = 338) were transferred to new cages and fed experimental diets *ad libitum* for 12 weeks. The experimental diets were: D12450J, 10% refined low-fat diet (LFD), catalogue number E157451-047; and D12492, 60% refined high-fat diet (HFD), catalogue number E15742-347 (Ssniff Spezialdiäten, Germany). Please consult Supplementary Fig. S1 and Table S1 for a flow chart of mouse distribution and compliance with downstream procedures.

Mice were handled in alternating order for all experimental procedures. All procedures were performed each day at approximately the same time in all housing units. Two units (3 and 4) shared animal caretakers, i.e., on any given day, mice at unit 3 were first managed, followed by mice at unit 4 (animal caretakers changed clothes in-between). Experimental design is graphically illustrated in Figure 1A and 1B.

### 2.3 Factorial design

A schematic overview of distribution, experimental variables, and tested factors are presented in Figures 1A and S1. Briefly, 26 reference mice (13 males and 13 females; eight group-housed and five single-housed per sex) were kept on a chow diet at the vendor's breeding unit. The remaining mice were randomly distributed into equal groups fed purified diets, either LFD or HFD for 12 weeks. Because mice are known to have an ambient temperature preference at ∼ T_30_ rather than ∼ T_22_ [13], a sub-experiment replicated in two different units was conducted to compare the influence of temperature on DIO and glucose regulation. We further studied the impact of sex by comparing 78 male and female mice, fed either LFD or HFD, replicated in three different units. In addition, we investigated the interaction between social isolation (single-versus group-housing) and remaining factors in relation to metabolic outcomes.

### 2.4 Intraperitoneal glucose tolerance test

For the first cohort of mice, an intraperitoneal glucose tolerance test (ipGTT) was performed 7 weeks after the introduction of the experimental diets. Mice were fasted for 5 h in AM (7–12) in clean cages only containing a semi-transparent plastic shelter, bedding, and water before glucose injection (2 g glucose/kg body weight). Blood glucose was measured in tail vein blood immediately before the procedure and 15, 30, 45, 60, 90, and 120 min after the glucose bolus, using an automated glucose analyzer (Bayer Contour, USA). The incremental area under the curve (iAUC) was calculated using GraphPad Prism software.

### 2.5 Fasting insulin

For the second cohort of mice, insulin was measured in plasma samples collected by tail vein after 7 weeks of feeding, akin to the ipGTT in the first cohort, and a 5-h fast (7–12) in clean cages only containing a semi-transparent plastic shelter, bedding, and water, using an electro-chemiluminescence mouse/rat insulin assay (Mesoscale Diagnostics, USA) according to the manufacturer's protocol.

### 2.6 Indirect calorimetry

For the second cohort of mice, at weeks 1, 6 and 11, mice were moved from standard housing cages to Calocages (PhenoMaster, TSE Systems, Germany) 72 h before transfer to the associated PhenoMaster cabinets to minimize the confounding effect of transfer-related stress. We further allowed a 24 h acclimatization period post-transfer to the cabinets before acquiring recordings using 15 min intervals for 48 h. Oxygen consumption (VO_2_) and carbon dioxide production (VCO_2_) were measured. Respiratory exchange ratio (RER) and energy expenditure (EE), corrected for body weight, were calculated using the measurements of O_2_ consumption and CO_2_ production (VCO_2_/VO_2_) [17] and (3.9 cal/L x VO_2_ + 1.1 cal/L x L VCO_2_) [18], respectively. Percent relative cumulative frequency (PRCF) curves were calculated as previously described [19]. Briefly, individual data points (∼192 per mouse, recorded every 15 min for 48 h) were sorted (from smallest to highest) to generate cumulative frequency sequences (frequency of each data increment), which was subsequently converted to PRCF. EC50 and hill slope were calculated using nonlinear regression of sigmoidal dose–response (variable slope) curve. Notably, EC50 is positively correlated with TEE and RER, whereas hill slope is *inversely correlated* with flexibility (i.e., variance between light and dark phase).

### 2.7 Corticosterone

Total fecal samples from a single cage over a period of 24 h was collected, sorted out of the bedding and stored at −20 °C. Corticosterone metabolites were extracted from fecal samples with 96% ethanol overnight, centrifuged for 20 min at 2,000 g twice and then 15 min at 10,000 g, only supernatant being kept in between centrifugation steps, all performed at room temperature. Final supernatant was then evaporated in borosilicate vials. Samples were reconstituted in PBS and corticosterone was measured using a commercial ELISA (DRG Diagnostics, EIA-4164) according to manufacturer's instructions [20].

### 2.8 Necropsy

Non-fasted mice (starting at 8 AM) were anesthetized using 2.5% isoflurane, bled by cardiac puncture, and euthanized by cervical dislocation before organ removal. Organs were carefully dissected and weighed by trained personnel strictly adhering to an established in-house standard operating procedure. To minimize the risk of interpersonal variation, and thus enhance experimental reproducibility across units, we deliberately designed a simplified dissection protocol, where only liver and the easily definable and accessible gonadal white adipose tissue (gWAT) depot were dissected. All tissue weight were recorded as wet weight.

### 2.9 Gut microbiota composition

Publicly available gut microbiota composition data [11,21] from 72C57BL/6 J mice arriving in 4 separate batches (1 batch/week) from the same vendor (Jackson Lab, North America), housed three mice/cage in six cages/batch (n = 18 per batch) were used to analyze the impact of batch and cage on gut microbiota composition. Briefly, feces from each batch were collected one week apart on dry ice, stored at minus 80 °C until downstream processing. All 72 samples were subsequently processed simultaneously (DNA extraction, library generation and sequencing). Bacterial DNA was extracted using the NucleoSpin 96 soil kit (Macherey–Nagel) following the manufacturer's instructions. 16 S rRNA gene was amplified over 25 cycles using primers for the V3–V4 and sequenced on an Illumina MiSeq desktop sequencer using the MiSeq Reagent Kit V3 (Illumina). Principal coordinate analysis (PCoA) of microbiota composition was carried out using unweighted UniFrac distances.

### 2.10 Statistical analysis

Analysis of the influence of experimental factors on metabolic outcomes was performed in R. Mixed linear models (LMMs) were fitted using the glmmTMB package (version 1.1.10) with experimental diet, cage type, housing temperature, group-vs single-housing, sex and first-order interactions with experimental diet as fixed effects. Only variables included in the respective analysis was adjusted for. Housing units were modelled as random effects. We computed the estimate for the average fold change compared to a typical facility (the median) as Exp (SD^2^_Fac_/2) and subsequently compute the 95% confidence interval for the facility SD. Residual's standard deviations (i.e., the mouse-to-mouse variation) were allowed to differ between single- and group-housed mice. All metabolic outcomes were log-transformed, and model appropriateness was assessed by plots of fitted values vs residuals. Marginal means were used to assess the main effect of each experimental factor (emmeans package, version 1.10.7). Interaction effects were tested on relevant subsets of data (aligning with illustrative figures), using similar LMMs and p-values were adjusted with Holm's method to control the family-wise error rate over 32 tests.

Sample size calculations were carried out for a fold change of 1.5, using t-tests on log-scale with equally many mice in each treatment group. This was done separately for single- and group-housed mice, using the residual coefficients of variation (CVs) from the LMMs. The results apply to studies carried out at a single housing unit. Furthermore, sample size calculations were carried out for studies carried out at three different units with equally many mice and both treatments at all units. This was done with simulations, using estimated residual CVs and housing unit CVs.

No samples were excluded from subsequent analysis. Still, three and six values are missing from the GTT and necropsy graphs, respectively, as the measurement could not be recovered (e.g., if a mouse unexpectedly succumbed).

## 3 Results

### 3.1 The impact of experimental variables on metabolic phenotype in diet-induced obese mice

**Figure 2:**
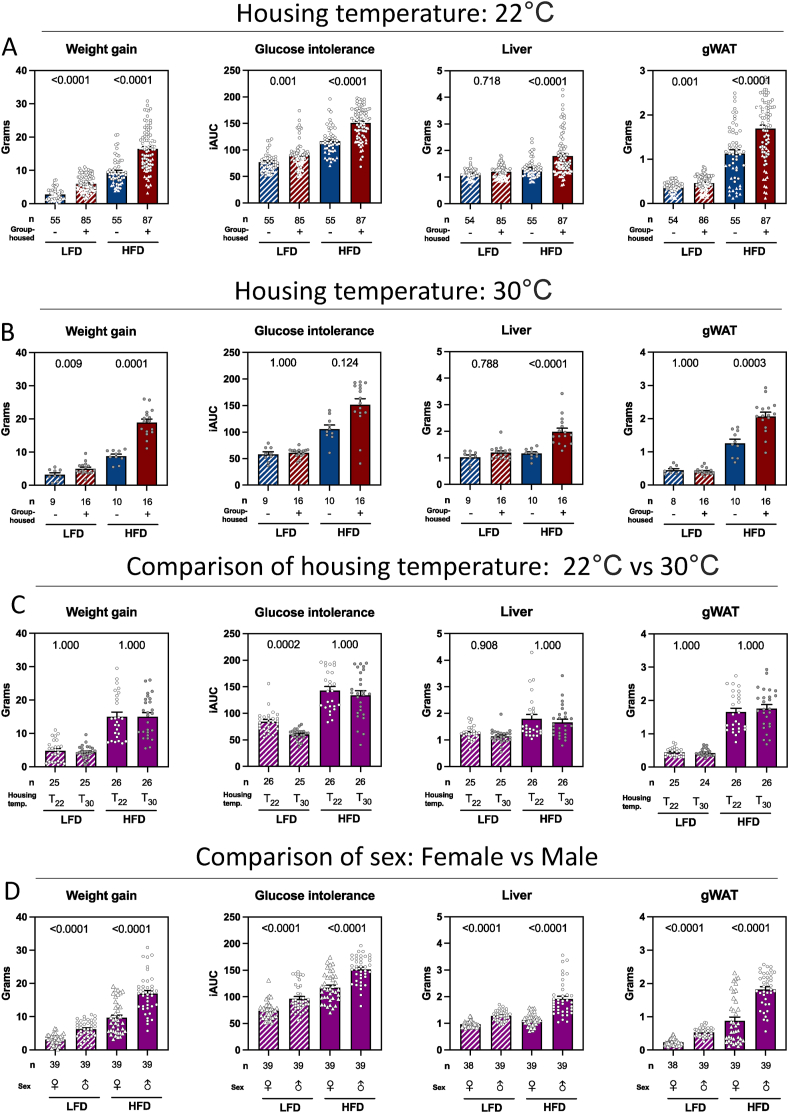
**Experimental variables differentially impact DIO.** A: Weight gain after 12-weeks, glucose intolerance at week 7, liver and gWAT weight at termination, separated in diets and single vs group-housing as indicated. B: As in A but from mice housed at thermoneutrality. C: Male mice housed in conventional cages at either T_22_ or T_30_ (2 units, 1 and 2) D: Male and female mice housed at T_22_ in IVC cages (3 units, 3, 4 and 5). Data shown as individual points, average and SEM. Blue bars represent single-housed mice, red bars group-housed mice, and purple bars represent a combination of single- and group-housed mice. Dashed patterns indicate LFD, and solid bars indicate HFD-fed mice. White dots indicate housing temperature of 22 °C, and grey points represent housing temperature of 30 °C. Round points indicate males, and triangles indicate females A: n = 63–103, B: n = 9–16, C: n = 24–26, D: n = 38–39, per condition as indicated under each bar. iAUC: incremental area under the curve.

We applied a Gaussian LMM on log-transformed data to define the hierarchy of experimental variation and used the estimates from the model to quantify the biological impact of tested variables (Figure 1C). Although experimental diet (HFD vs LFD) was the strongest driver of metabolic discrepancies, group-vs single-housing was generally comparable to sex (males versus females) and a more powerful regulator of the elicited host response to DIO and glucoregulatory disturbances than cage type (IVC vs conventional), housing temperature (30 vs 22 °C), and housing unit. Only liver weight deviated substantially from this pattern, as male mice presented ∼45% bigger livers (fold change of 1.44) than their female counterparts, while the impact of diet and group-vs single-housing each amounted to ∼20% (fold change of 1.20 and 1.21, respectively). Cage type did not affect any outcome. In contrast, housing unit had a significant impact on weight gain, liver and gWAT weight, although the effect size was minimal. As such, HFD-fed single-housed mice exhibited diminished *i*) weight gain, *ii*) enhanced glucoregulatory capacity *iii*) decreased liver and *iv*) gWAT mass, compared to their group-housed counterparts at 22 °C. (Figure 2A). While single-housing mostly affected gWAT and liver weight, as well as glucose intolerance, in HFD-fed rather than LFD-fed mice (Figure 2A), we found diminished weight gain as a consistent trait across diets.

Notably, the impact of housing strategies was replicated at both room temperature (22 °C, T_22_) and thermoneutrality (30 °C, T_30_), indicating that the attenuated susceptibility to DIO and associated metabolic disturbances in single-housed mice was indeed caused by single-housing *per se*, and thus not loss of heat as a function of affected nesting behavior (Figure 2A–B). Still, since housing temperature has attracted considerable attention as a potent modulator of phenotypic traits in rodents [13], including in the fields of cardiology [22], immunology [23], metabolic dysfunction-associated steatotic liver disease (MASLD) [14], and because persisting differences between group- and single-housed mice do not exclude an impact of temperature *per se*, we next measured the interaction between temperature and diet to quantify the experimental variation. Male mice were equally distributed between T_30_ and T_22,_ and the experiment was reproduced in two independent units (n = 13 mice per unit, per temperature, per diet, for a total of 104 mice). In contrast to glucose tolerance, weight gain, liver and gWAT mass were not affected by ambient temperature (Figure 2C). The apparent improved glucose tolerance in mice housed at thermoneutrality was driven by a significant change in LFD-fed but not HFD-fed mice (Figure 2C).

We next assessed the impact of sex on the outcomes (3 units, n = 13 mice of each sex, per unit, per diet, for a total of 156 mice). We found female mice, on LFD or HFD, to exhibit improved metabolic parameters compared to their male counterparts on all assessed variables (Figure 2D). Despite this obvious sex-bias, female mice generally responded to HFD diet intervention displaying increased body weight, gWAT weight, and glucose intolerance. Still, female mice did not develop enlarged livers in response to HFD, contrasting their male counterparts (Figure 2D).

Body weight differences were not fully explained by caloric intake alone. Paradoxically, group-housed female mice consumed less energy than single-housed females yet exhibited greater weight gain (Figure 2A, S2A). This sex-specific pattern contrasted with male mice, where group-housed males consumed more food than single-housed males, aligning with their heightened susceptibility to DIO. Additionally, housing temperature modulated feeding behaviour: mice at thermoneutrality, where thermoregulatory demands are abolished, displayed reduced food intake compared to those exposed to mild thermal stress (Figure 1C). These findings underscore the complex interplay between housing conditions, sex, and metabolic efficiency in shaping energy balance.

### 3.2 Variance is not increased by hierarchical organization in group-housed mice

**Figure 3:**
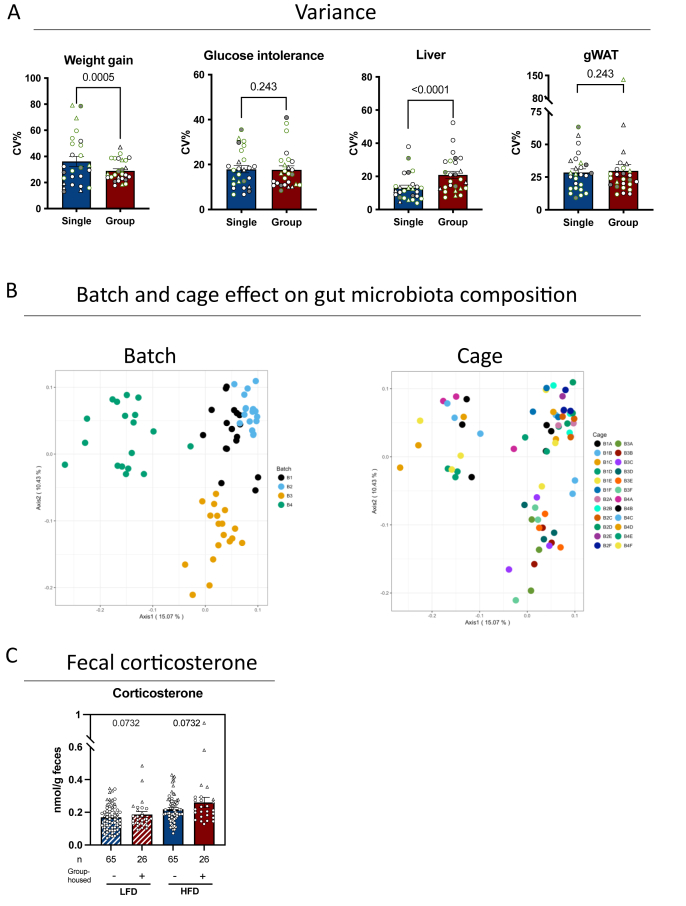
**Impact of housing on variance, microbiota and stress.** A: Intragroup variation on each measured outcome presented by the CV% per condition. Each point represents the variation between mice in a single category in each facility for a total of 28 for weight gain, liver, and gWAT and 26 for glucose intolerance. Data shown as individual points, average and SEM. Each data point per bar represents the CV% from either 8 (group-housed) or 5 (single-housed) mice. Circles = males, Triangles = females, White = T_22_, Grey = T_30_, LFD = outer line green and HFD = outer line black. B: Principal Coordinate Analysis (PcoA) unweighted UniFrac colored by either batch shipment (4 batches, n = 18 per shipment) or cage (24 cages, n = 3 per cage). C: Corticosterone in fecal samples. Each point represents a cage, thus, for group-housed cages, each dot is an average for 4 mice whereas for single-housed mice, each dot is a single animal.

The inevitable formation of a hierarchy between group-housed male mice is generally assumed to exacerbate group/cage variation. To address whether group-housing induces higher variability (cage effects), we calculated the relative coefficient of variation percentage (CV%) within single- and group-housed mice and compared ranks (Figure 3A). Each dot represents variation for one combination of experimental variables; for example, eight group-housed male mice fed HFD were housed in two IVC cages at thermoneutrality in unit 1. Thus, each data point represents the CV% from either 8 (group-housed) or 5 (single-housed) animals. We found the intragroup variation of visceral fat accretion and glucose intolerance to be remarkably similar between single and group-housed mice, while weight gain varied more in single-housed mice (Figure 3A). In contrast, intra-group variation of liver weight was more pronounced in group-housed mice than it was in their single housed counterparts (Figure 3A).

Yet, even if hierarchy does not systematically enhance intragroup variation, it may come at a noteworthy price for host-diet-microbiome research in DIO, considering the coprophagic nature of mice potentially converting individual microbiomes to a cage microbiome [24]. To address this concern, we sourced publicly available data from our previous publications [11,21], where we housed male mice three by three (n = 24 IVC cages, n = 72 mice) and arrived in four different batches (one week apart) from a single vendor, and geographical location (North America). All mice were acclimatized on a purified LFD and sampled two weeks after arrival. While microbiota composition did not separate by cage ID, hence pointing against a single cage microbiota, our analysis revealed that the mouse batch was a pronounced driver of baseline gut microbiota composition (Figure 3B, S3A). Most of these changes were driven by low abundant species (unweighted unifrac distances, Figure 3B), while top 20 most abundant species remained relatively stable in three out of four batches (Fig. S3A). Based on this, we conclude that baseline microbiota composition must be considered as potential a source of variation in results using repeated data from commercially raised mice, whereas potential cage effects appear negligible.

We next asked if housing conditions modulated stress, either from social deprivation in single-housed mice or hierarchical social dynamics in group-housed counterparts. To assess chronic stress levels, we quantified 24-hour accumulated fecal corticosterone after eight weeks of DIO. Consistent with established sex differences [25] male mice exhibited approximately 50% lower corticosterone levels than females (Figure 1C). Dietary and environmental factors further influenced stress hormone levels: mice on HFD displayed elevated corticosterone levels compared to LFD or standard chow groups (HFD > LFD ≈ chow, Figure 1, Figure 3C, S3B), while thermoneutral housing (T_30_) significantly reduced levels relative to mild thermal stress (T_22_, Figure 1C). Surprisingly, group-housed mice displayed modestly higher corticosterone than single-housed mice across dietary conditions (Figure 1, Figure 3C, S3B). Collectively, these results indicate that while temperature, sex, and diet robustly modulate chronic corticosterone secretion, social isolation—contrary to our hypothesis—does not exacerbate stress responses compared to group housing after eight weeks.

To leverage our controlled setup and a considerable number of mice, we performed power calculations to determine appropriate sample size. While this is a requirement in clinical trials, it is often overlooked in preclinical research. This is unfortunate as it could minimize the number of faulty interpretations, hence enhancing reproducibility and potentially clinical translatability [26,27]. Thus, we calculated the number of mice necessary for a 1.5-fold change to be detected with 80% probability at significance level 0.05 between mice fed LFD vs HFD over 12 weeks in both single- and group-housed mice (Table 1). Aligning with the more pronounced diet-induced metabolic disturbances in group-housed mice, this housing condition decreased the number of mice needed to detect an effect for weight gain from 25 to 14. Minimal number of mice was in favor of single-housed mice for the other three parameters although numbers remained more similar between both conditions.

**Table 1:** Power calculation.

	Single-housed	Group-housed
Weight gain	25	14
Glucose intolerance	6	7
Liver weight	5	8
gWAT weight	13	16

Number of mice per group required.

### 3.3 Single-housed mice exhibit altered diurnal activity patterns and increased energy expenditure

**Figure 4:**
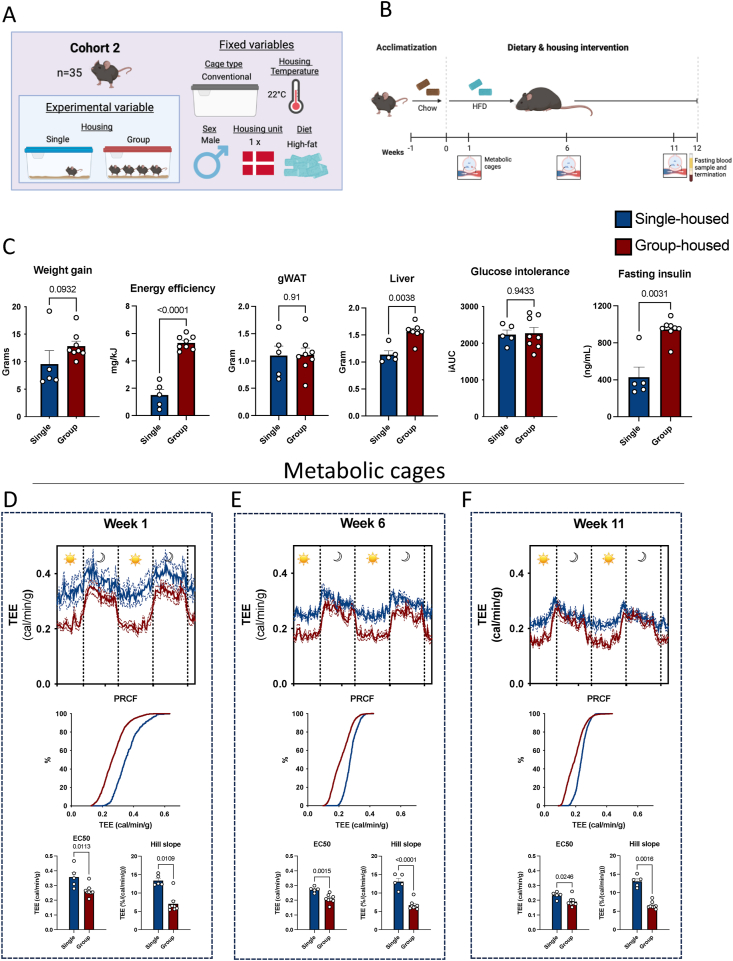
**Socially deprived mice exhibit altered circadian rhythm and increased energy expenditure** A: Visual representation of the different fixed and experimental variables. B: Experimental timeline of the second cohort of mice. C: Body weight gain, energy efficiency, liver and gWAT weight, glucose intolerance (iAUC), and 5 h fasting insulin concentration after 12 weeks of HFD-feeding. Energy expenditure, relative cumulative frequency, and hill slope at weeks D: 1, E: 6, and F: 11 as indicated. EC50 and hill slope were calculated by nonlinear regression of the sigmoidal dose–response curve. Data shown as individual points, average and SEM. Blue represent single-housed mice, red represents group-housed mice. Grey and light areas indicate dark and light periods, blue represents single-housed (n = 5) and red represents group-housed (n = 8). TEE: Total energy expenditure. PRCF: Percent relative cumulative frequency.

The above-described discrepancies between group- and single-housed mice prompted us to investigate how housing strategy could drive experimental variability. To this end, we initiated a new HFD experiment in male mice (Figure 4A–B). These results corroborated that decreased weight gain was explained by reduced energy efficiency measured as weight gain per unit of energy consumed, in single-housed mice compared to group-housed mice (Figure 4C).

To identify factors contributing to the observed metabolic differences, we next analyzed total energy expenditure (TEE) and respiratory exchange rate (RER) at weeks 1, 6, and 11 post initiation of diet intervention. Single-housed mice exhibited increased 48 h energy expenditure combined with a more static activity pattern as observed by an increased Hill slope (Figure 4D-E-F). The increased energy expenditure was predominantly driven by single-housed mice burning considerably more energy during the light period than their group-housed counterparts. Energy expenditure in single-housed mice also varied less by light-phase compared to group-housed mice, indicating that single-housing interferes with normal diurnal rhythms. The change in behavior was established already 1 week after social deprivation and persisted until the last measure at week 11, albeit slowly converging towards group-housed animals over time (Figure 4D-E-F). Similar traits were shown for substrate utilization, where single-housed mice metabolized more fat than their group-housed counterparts, as indicated by lower RER, particularly during the first 6 weeks (Fig. S4). Our results thus suggest that single housing contributed to decreased metabolic effects of HFD feeding through several paths, including increased energy expenditure combined with a shift in substrate utilization demonstrating a disturbed diurnal activity pattern.

## 4 Discussion

Here, we investigated the impact of several experimental variables on the DIO phenotype in C57BL6/JBomTac mice, using a multi housing-unit intervention. Notably, while sub strain [28] and vendor [29] may impact metabolic trajectory, C57BL6 mice on a J background, including the JBomTac [30], is widely recognized as excellent models for DIO [29]. Our setup was designed to control unit-to-unit variation to demonstrate reproducibility and quantify the impact of an ideal setup across units. We measured metabolic outcomes that could be performed similarly at all experimental units with minimal interpersonal variation. Logistical constraints did, however, prevent full factorial inclusion of all variables across experimental units (e.g., female cohorts were limited to three units, and thermoneutral housing was tested exclusively in males across two units). To address variability between experimental units and unbalanced design factors, we incorporated sex as a fixed effect in LMMs, ensuring robust cross-condition comparisons. The absence of e.g., female data under thermoneutral conditions was thus accounted for statistically through model design. As both diet and vendor are well-known variables for host metabolism [7,11], we carefully controlled the run-in diet – i.e., “meal packs” from the vendor, consult material and methods for details – and only used a single sub-strain of mice from one vendor – all shipped simultaneously and from the same breeding unit – to capture the impact of technical variables better. An exception to this controlled design was our inclusion of publicly available 16 S rRNA sequencing data to investigate cage- and batch-dependent microbiota variation. While these data originated from a different mouse sub strain and vendor than our primary cohort, they provide critical mechanistic insights into microbial community dynamics. Our analysis revealed that cage-level effects on microbiota composition were negligible, even in coprophagic mice, whereas batch-to-batch variation significantly influenced low-abundance taxa. This conclusion is further supported by an independent analysis of 720 samples from our main cohort across six facilities and three diets (LFD, HFD, chow). Although raw sequencing data from this cohort are unavailable due to a server malfunction, processed results demonstrating negligible cage effects can be acquired upon request.

Including technical variables via LMMs enabled us to estimate the effect of experimental variables on the metabolic outcomes while adjusting for housing units and cage type. While weight gain and gWAT weight were highly affected by experimental variables, glucose intolerance and liver mass overall had a lower effect size. Generally, diet, sex, and single-vs group-housing were the major drivers of within-facility experimental variation. Other technical variables, such as cage ventilation and housing temperature only had negligible impact on measured outcomes, while housing unit did exhibit small, yet significant, effects, except on glucose intolerance. Our interpretation of this finding is that despite nominal differences in absolute numbers between facilities (e.g., absolute body weight in a certain group), the overall phenotype and thus conclusion is fully recapitulated across housing units if the experimental setup is carefully controlled (i.e., same relative differences between groups across units). Although several of the included variables, such as sex and diet, have previously been described to affect host physiology [31], a significant advancement of our work is to rank these by effect size and replicate the findings across housing units and countries simultaneously.

A consistent observation across experiments was reduced weight gain in single-housed mice. Contrary to expectations, we detected modestly lower 24-hour accumulated fecal corticosterone in single-housed animals compared to group-housed counterparts. This finding initially appeared paradoxical, as Muta et al. reported an acute stress response (elevated corticosterone) in mice isolated for 4 days relative to pair-housed controls [32] - a phenomenon that aligns with the transient disruption in energy expenditure we observed one week after single housing (Figure 4D). Further supporting this temporal dynamic, Kamakura et al. documented elevated corticosterone levels in single-housed mice during the first two days post-isolation, which normalized, or even reversed, by day 7 [33]. Together, these studies suggest that single housing induces acute stress responses that diminish over time, potentially attenuating early DIO progression while seemingly sparing long-term hypothalamic-pituitary-adrenal (HPA) axis activation under chronic conditions [25]. These findings are further corroborated by Smolensky et al., who recently reported comparable serum corticosterone levels but reduced body weight gain in both male and female mice single housed for 4 weeks compared to their group housed counterparts [34].

This hormonal consistency contrasts with the striking metabolic divergence observed in our study. Thus, we found that reduced weight gain observed in single-housed mice was primarily driven by increased energy expenditure and decreased energy efficiency. Notably, these effects were independent of ambient temperature, as single- and group-housed mice exhibited similar weight trajectories under both mild thermal stress (T_22_) and thermoneutrality (T_30_). Together, these results support the conclusion that altered diurnal activity patterns and metabolic inflexibility alone were sufficient to mediate the anti-obesogenic effects of single housing. While this finding may seem surprising, considering that group-housed male mice a prone to hierarchical fallouts and chasing behaviour, our data indicate that such unwarranted behaviours were negligible compared to the likely stress-induced increase of movement by single housing – at least in relatively short-term (<12 weeks) experiments. Still, future studies integrating high-resolution calorimetry, activity monitoring, and dynamic temperature modulation are essential to comprehensively delineate the interplay of temperature, sex, and housing conditions (single vs. group) on metabolic trajectories under varying experimental conditions.

Along these lines, as housing temperature is gaining traction as a modifiable variable on at least metabolic inflammation, but not insulin resistance [13,35], we investigated the impact on reported outcomes. To this end, thermoneutral housing had a modest impact on metabolic phenotype, restricted to glucose regulation in LFD-fed mice. The lack of difference in glucose tolerance observed between HFD-fed mice at thermoneutrality and their room temperature housed counterparts in our and *Tian* et al.*‘s* studies [35] contrasts the findings by *Giles* et al. [14]. A potential explanation of this dichotomy could be experimental duration, and thus the severity of the model, as the latter study was conducted over 24 weeks to allow MASLD development; a potent driver of dysregulated glucose homeostasis [36,37]. Humidity is another environmental factor that has been shown to impact obesity development in mice [33], thus, it was specifically controlled for in this study to avoid an additional variable effect.

Lastly, it is noteworthy to mention strategies that can be used to mitigate fighting within group-housed male mice, as it can become an issue that significantly affects metabolic outcomes. From our experience, fighting behaviour is significantly decreased if male mice are ordered, delivered, and separated in their final group-housed cage at an early age, such as 4 weeks. Furthermore, avoiding the smell of female mice by using individually ventilated cages and by avoiding opening male cages in a room where female mice have been also decreases intra-cage fighting. Other potential stressors such as a stressed handler, lack of enrichment, vibrations (e.g., construction work), loud noises, or constant noises during daytime, when mice are meant to sleep, as well as strong smells like perfume and blood, should also be kept at minimal levels [38]. While these conditions must be experimentally addressed in future studies to decipher their individual contributions, it is our anecdotal observation from performing DIO studies with >10,000 mice in multiple facilities, countries, and continents, that above-mentioned recommendations diminish fighting behaviour and intragroup variations. Thus, until such an experiment can be formally performed, we recommend these actions as general guidelines.

In conclusion, this study provides a systematic and comprehensive ranking of common experimental variables affecting metabolic outcomes in murine DIO research. Our data confirm that diet is the principal driver of metabolic phenotype, with sex also exerting a substantial influence. Among the experimental variables tested, housing conditions (single-vs. group-housing) emerged as the most impactful controllable factor after diet and sex, consistently shaping weight gain, adiposity, and glucose regulation across multiple research units. These findings underscore the necessity of transparent reporting and careful consideration of housing strategy in preclinical metabolic studies to enhance reproducibility and interpretability.

## CRediT authorship contribution statement

**Béatrice So-Yun Choi:** Writing – original draft, Visualization. **Jacob Bak Holm:** Writing – review & editing, Investigation, Formal analysis. **Asker Brejnrod:** Writing – review & editing, Formal analysis. **Even Fjære:** Writing – review & editing, Investigation. **Zhongkui Xia:** Writing – review & editing, Investigation. **Marie-Louise Allingbjerg:** Writing – review & editing, Formal analysis. **Ida Søgaard Larsen:** Writing – review & editing, Investigation, Formal analysis. **David Møbjerg Kristensen:** Writing – review & editing, Investigation. **Morten Dall:** Investigation. **Lene Secher Myrmel:** Writing – review & editing, Investigation. **Janne Koch:** Writing – review & editing. **Niels Banhos Danneskiold-Samsøe:** Writing – review & editing, Investigation. **Otto Kalliokoski:** Writing – review & editing, Investigation. **Jonas T. Treebak:** Writing – review & editing. **Liang Xiao:** Writing – review & editing, Investigation. **Axel Kornerup Hansen:** Supervision, Investigation. **Helle Sørensen:** Writing – review & editing, Formal analysis. **Lise Madsen:** Writing – review & editing, Supervision, Methodology, Conceptualization. **Manimozhiyan Arumugam:** Writing – review & editing, Supervision. **Karsten Kristiansen:** Writing – review & editing, Supervision, Methodology, Conceptualization. **Benjamin A.H. Jensen:** Writing – original draft, Supervision, Methodology, Investigation, Funding acquisition, Formal analysis, Conceptualization.

## Financial support

The work in this manuscript was partly supported by Taconic Biosciences, Denmark, and Ssniff Spezialdiäten, Germany, for the delivery of mice and animal feed, respectively. BAHJ was supported by 10.13039/501100003554Lundbeck Foundation (Grant number: R232-2016-2425) and 10.13039/501100009708Novo Nordisk Foundation (Grant number: NNF17OC0026698, NNF21OC0066931). BSYC is supported by the BRIDGE – Translational Excellence Programme (bridge.ku.dk), funded by the 10.13039/501100009708Novo Nordisk Foundation (Grant number: NNF20SA0064340) as well as a postdoctoral fellowship from the 10.13039/501100000156Fonds de Recherche du Québec - Santé. AB was supported by Independent Research Fund Denmark (Grant 6111-00471B) and 10.13039/501100003554Lundbeck Foundation (grant R140-2013-13528). 10.13039/501100011747Novo Nordisk Foundation Center for Basic Metabolic Research is an independent Research Center, based at the 10.13039/501100001734University of Copenhagen, Denmark and partially funded by an unconditional donation from the 10.13039/501100009708Novo Nordisk Foundation (Grant number NNF18CC0034900 and NNF23SA0084103).

## Declaration of competing interest

The authors of this manuscript declare no conflicts of interest.

## Data Availability

Data is shared on public repository. DOI: 10.17632/8zjbyjj2s7.1.
