# Supplementary material for: Housing matters: Experimental variables shaping metabolism in obese mice
Source: Mol Metab. 2025 Jun 18;98:102190. doi: 10.1016/j.molmet.2025.102190 (PMC12269455; doi:10.1016/j.molmet.2025.102190)
Supplement: Multimedia component 1 [file mmc1.docx]

**Supplemental Table 1**

|  |  | **Housing status** | | **Sex** | | **Housing temperature** | | **Cage type** | | **Diet** | | |
| --- | --- | --- | --- | --- | --- | --- | --- | --- | --- | --- | --- | --- |
| **Unit** | **n** | **Single** | **Group** | **Male** | **Female** | **22°C** | **30°C** | **Conv.** | **IVC** | **LFD** | | **HFD** |
| **Breeding** | **26** | 10 | 16 | 13 | 13 | 26 | 0 | 26 | 0 | *Reference chow (26)* | | |
| **Unit 1** | **78** | 30 | 48 | 78 | 0 | 52 | 26 | 52 | 26 | 39 | 39 | |
| **Unit 2** | **78** | 30 | 48 | 78 | 0 | 52 | 26 | 52 | 26 | 39 | 39 | |
| **Unit 3** | **52** | 20 | 32 | 26 | 26 | 52 | 0 | 0 | 52 | 26 | 26 | |
| **Unit 4** | **52** | 20 | 32 | 26 | 26 | 52 | 0 | 0 | 52 | 26 | 26 | |
| **Unit 5** | **52** | 20 | 32 | 26 | 26 | 52 | 0 | 0 | 52 | 26 | 26 | |
| **Unit 6** | **26** | 10 | 16 | 26 | 0 | 26 | 0 | 26 | 0 | 13 | 13 | |
| **Total per column** | **364** | **140** | **224** | **273** | **91** | **312** | **52** | **156** | **208** | **169** | **169** | |
| **Total per variable** |  | **364** | | **364** | | **364** | | **364** | | **364 (338+26)** | | |
