# Supplementary material for: Housing matters: Experimental variables shaping metabolism in obese mice
Source: Mol Metab. 2025 Jun 18;98:102190. doi: 10.1016/j.molmet.2025.102190 (PMC12269455; doi:10.1016/j.molmet.2025.102190)
Supplement: Multimedia component 2 [file mmc2.pptx]

## Slide 1
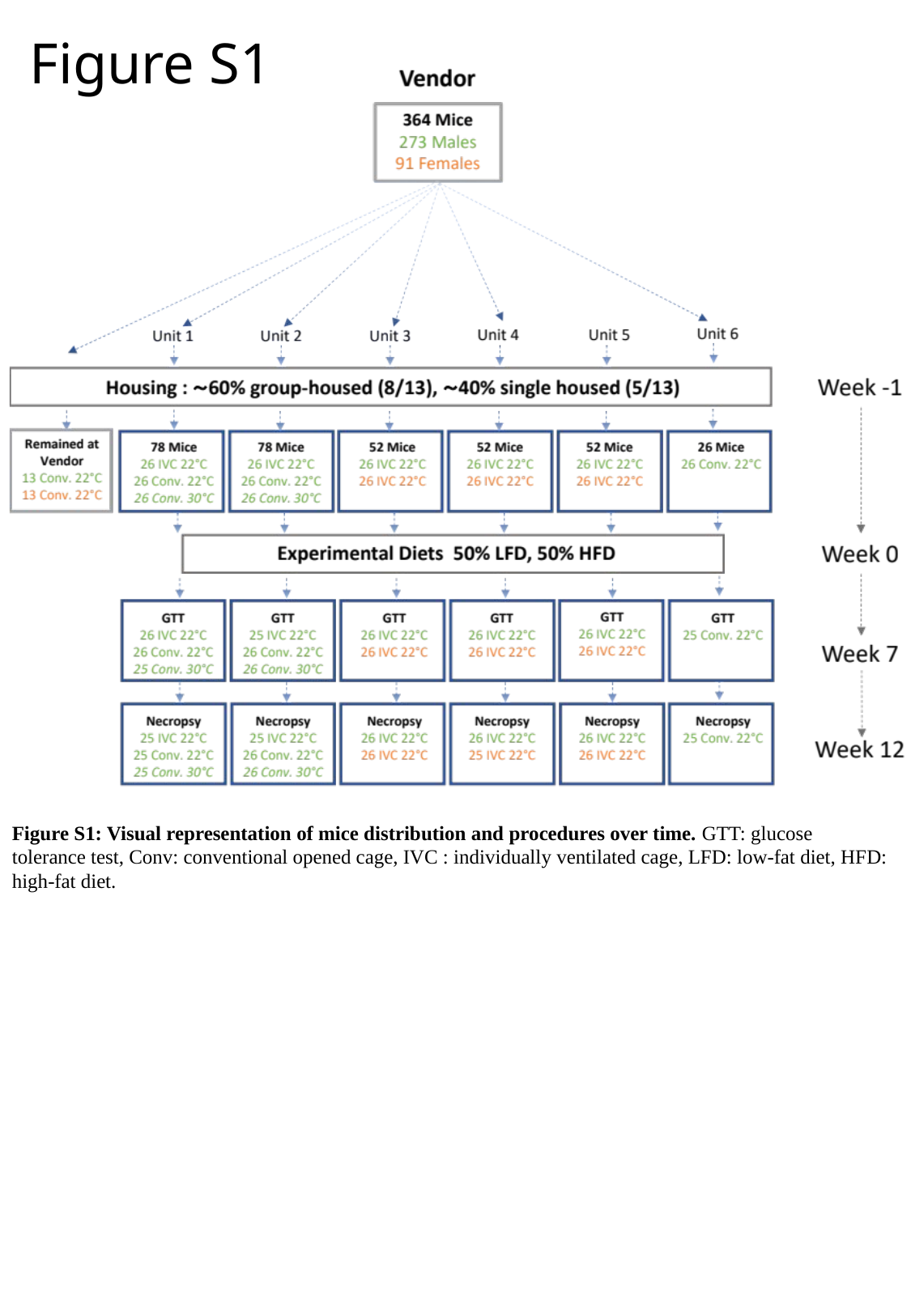

Figure S1
Figure S1: Visual representation of mice distribution and procedures over time. GTT: glucose tolerance test, Conv: conventional opened cage, IVC : individually ventilated cage, LFD: low-fat diet, HFD: high-fat diet.

## Slide 2
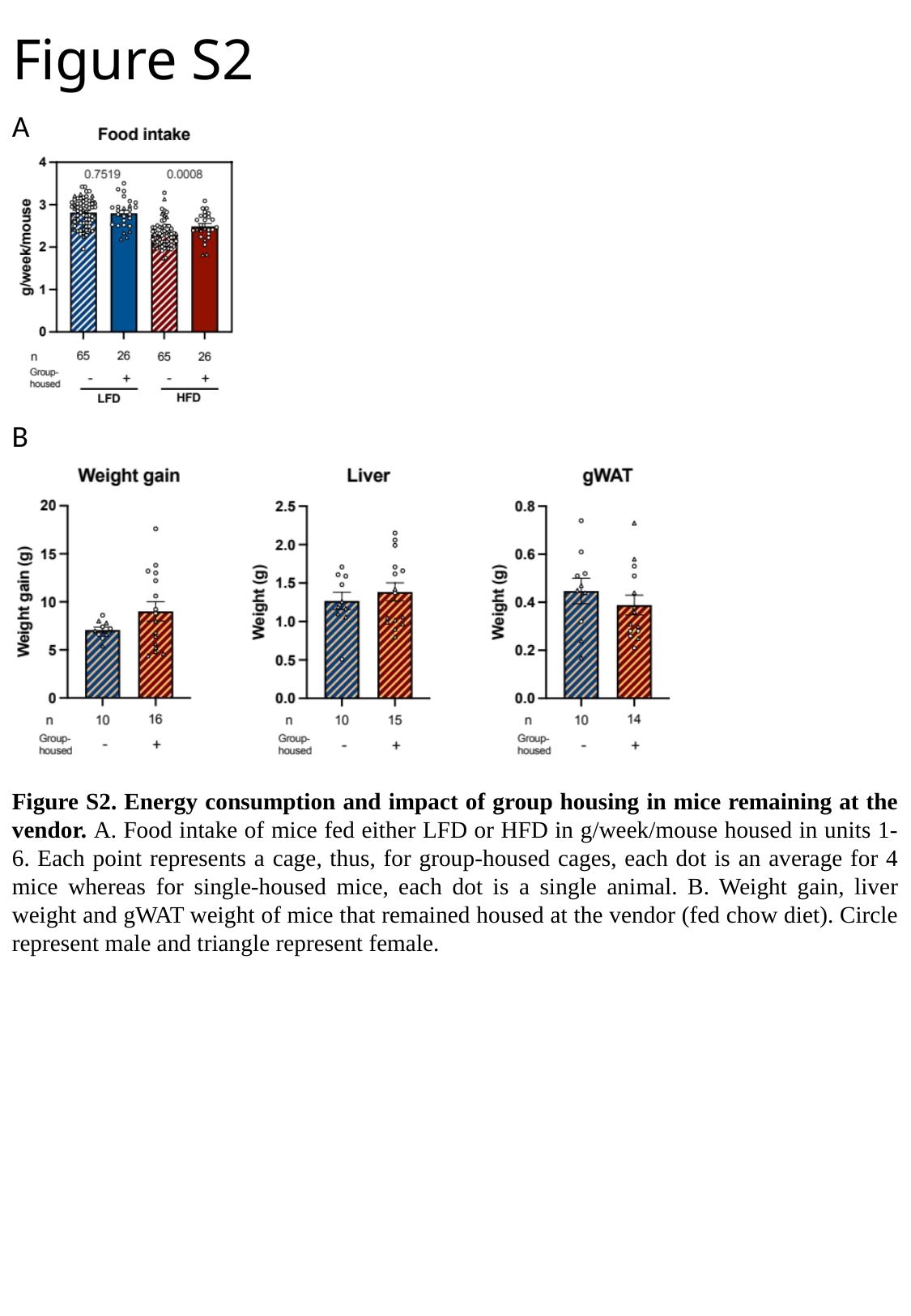

Figure S2
A
B
Figure S2. Energy consumption and impact of group housing in mice remaining at the vendor. A. Food intake of mice fed either LFD or HFD in g/week/mouse housed in units 1-6. Each point represents a cage, thus, for group-housed cages, each dot is an average for 4 mice whereas for single-housed mice, each dot is a single animal. B. Weight gain, liver weight and gWAT weight of mice that remained housed at the vendor (fed chow diet). Circle represent male and triangle represent female.

## Slide 3
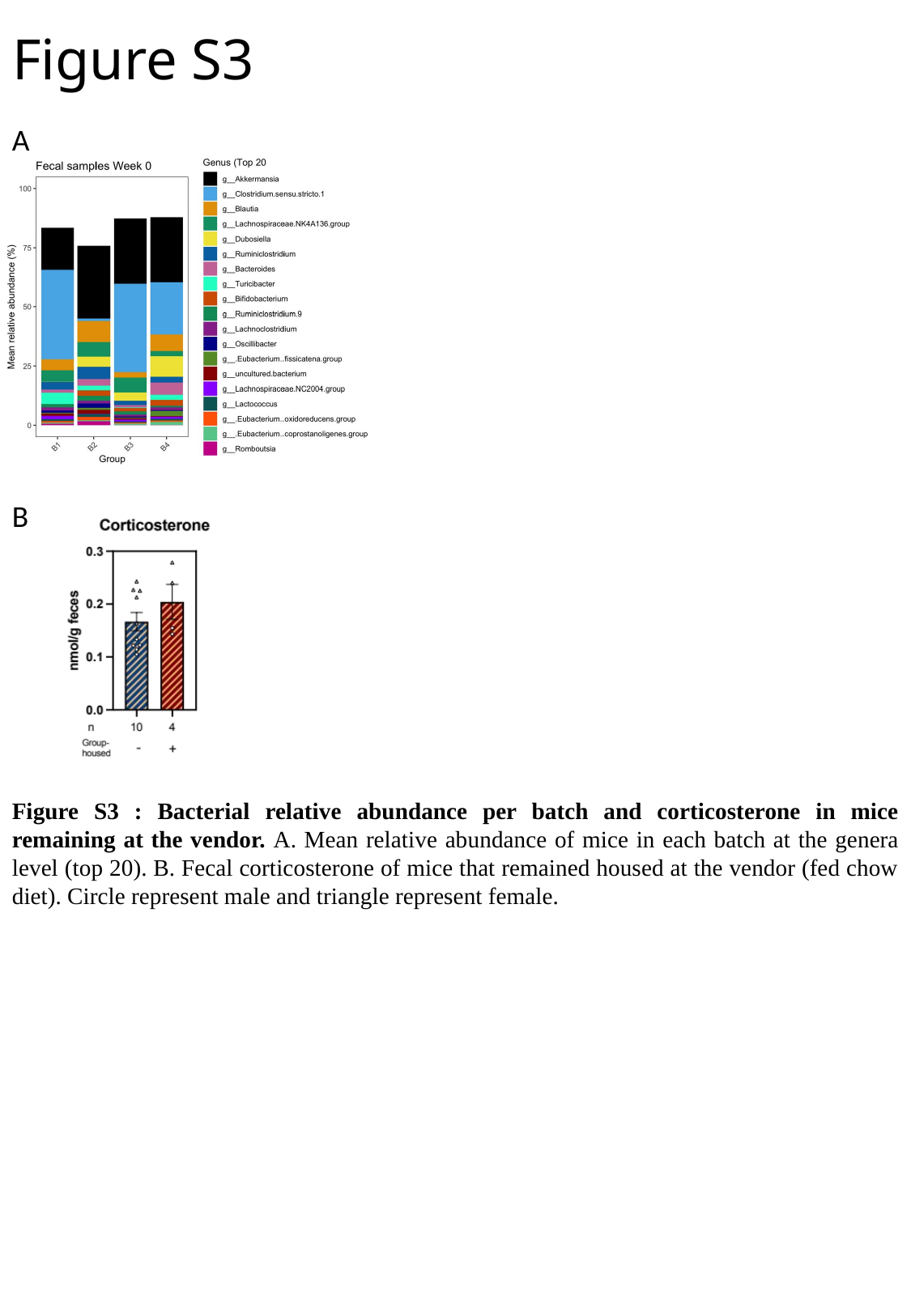

Figure S3
A
B
Figure S3 : Bacterial relative abundance per batch and corticosterone in mice remaining at the vendor. A. Mean relative abundance of mice in each batch at the genera level (top 20). B. Fecal corticosterone of mice that remained housed at the vendor (fed chow diet). Circle represent male and triangle represent female.

## Slide 4
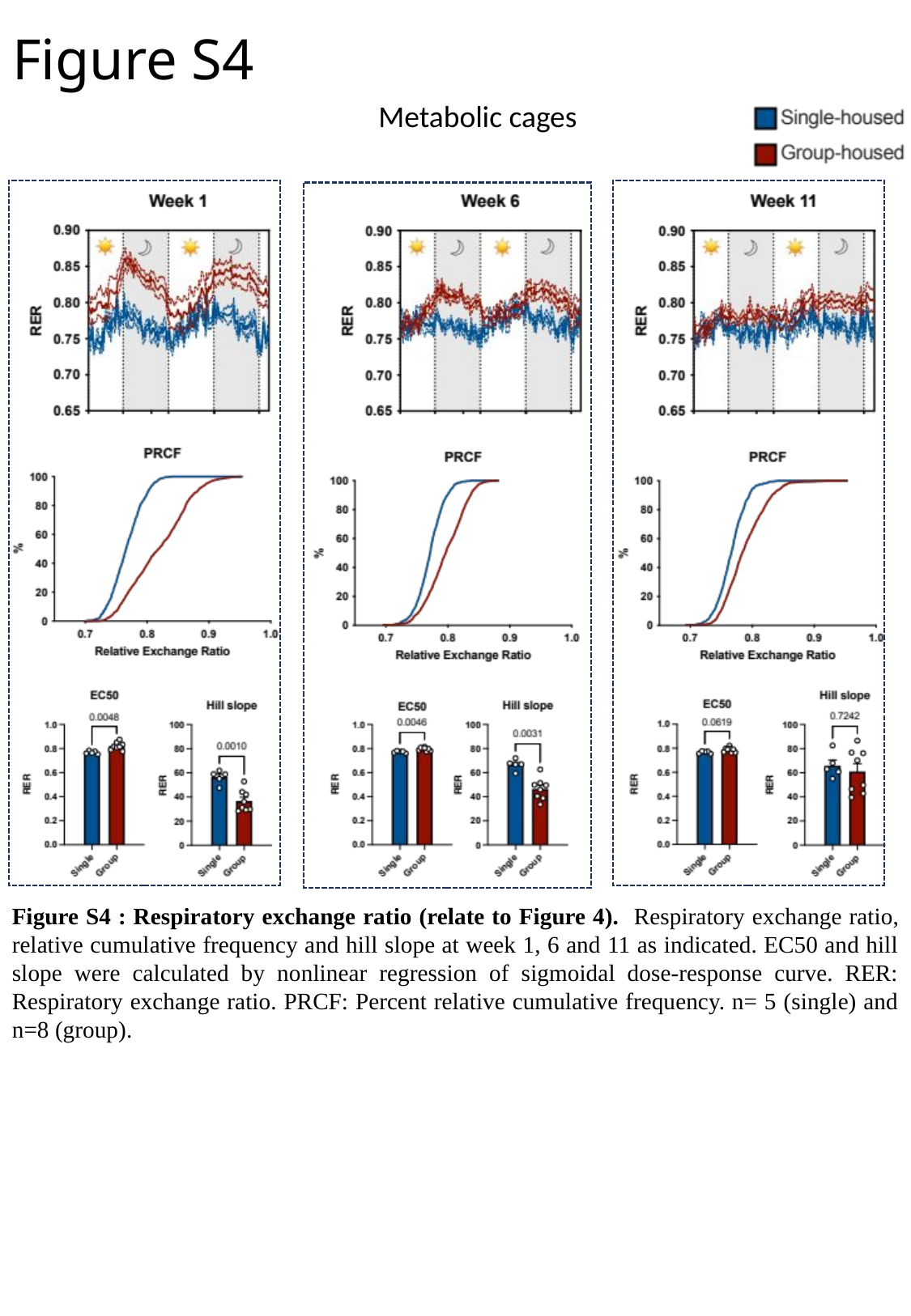

Figure S4
Metabolic cages
Figure S4 : Respiratory exchange ratio (relate to Figure 4). Respiratory exchange ratio, relative cumulative frequency and hill slope at week 1, 6 and 11 as indicated. EC50 and hill slope were calculated by nonlinear regression of sigmoidal dose-response curve. RER: Respiratory exchange ratio. PRCF: Percent relative cumulative frequency. n= 5 (single) and n=8 (group).
